# Quo vadis? – Medical education 2020 between politics and science

**DOI:** 10.3205/zma001011

**Published:** 2016-02-15

**Authors:** Sigrid Harendza, Martin R. Fischer, Götz Fabry

**Affiliations:** 1 Universitätsklinikum Hamburg-Eppendorf, III. Medizinische Klinik, Hamburg, Germany; 2 Klinikum der Universität München, Institut für Didaktik und Ausbildungsforschung in der Medizin, München, Germany; 3 GMS Journal for Medical Education, Editor-in-Chief, Erlangen, Germany; 4 Albert-Ludwig-Universität Freiburg, Abt. für Med. Psychologie, Freiburg/Brg, Germany; 5 GMS Journal for Medical Education, Deputy Editor, Erlangen, Germany

## Editorial

With its so-called Masterplan for Medical Education (“Masterplan Medizinstudium”) 2020, the German government together with the federal states intends to develop measures with the aim of enabling a more targeted selection of study applicants, increase the orientation towards practice, and strengthen the primary care aspects throughout the studies [Shaping Germany's Future, coalition treaty between CDU, CSU and SPD, retrieved 30.1.2016]. In the internet, one can already find a variety of statements as well as suggestions for according measures by the Medical Faculty Association (MFT), German Medical Students' Association (bvmd), German Medical Association, German physicians' union (Marburger Bund, Hartmannbund), as well as some societies within the Association of Scientific Medical Societies in Germany (AWMF). These various perspectives paint a multi-faceted and partly contradicting image of recommendations. However, what most of these recommendations have in common is that they are not based on scientific evidence.

### More targeted selection of study applicants

Hardly anyone would argue that the final school grade (Abitur) and the test for medical studies (“Test für Medizinische Studiengänge” [TMS]) are legally secure and a practical means for the selection of students. Moreover, hardly anyone would seriously claim to know of a selection procedure which enables reliable predictions whether a student will become a “good physician” after six or more years. Thus, the validity of existing selection procedures can solely be determined with the help of surrogate parameters. As outlined in this issue [1], scientific evidence has shown, for instance, that the TMS allows for a differentiation of potentially successful and less successful students on the basis of specific grades in the Abitur exam. On the contrary, the Abitur grade alone correlates with study performance and study delays but not with the completion of studies within the designated study period [2]. 

There is often the objection that study performance (particularly if understood as mainly cognitive requirements in the sense of knowledge-based exams) only display a part of the competencies and personality traits that professionals in the medical field need to have. Against this backdrop, selection procedures such as the Multiple-Mini-Interview (MMI) have rapidly been spreading, as they include an assessment of psychosocial and communicative competencies regarded as crucial for medical professionals. Supporters of such procedures, however, need to take into account that only a single study – out of 66 published studies on MMIs – was able to predict study behavior and grades in the final exams to an extent that may be considered noteworthy. The construct validity of many other MMIs appears to be insufficient [3] in addition to this procedure being rather costly and time-consuming in general.

Further selection criteria applied by German universities are weighted individual scores from the Abitur exam, subject-specific aptitude tests, type of vocational training, preferred location for studies, or outcomes of a selection interview [Hochschulstart, retrieved 30.1.2016]. Other countries, such as The Netherlands, have established some kind of weighted lottery based on Abitur grades in which study applicants with a less-than-perfect Abitur also have a chance to get admitted to studying medicine at the university [Studium in den Niederlanden, retrieved 30.1.2016]. For a more targeted selection of study applicants as proposed in the “Masterplan Medizinstudium 2020”, it appears mandatory to define the purpose of this selection first: Is the central point to allow for a study aptitude assessment that is more specific than the Abitur grade? Should students be selected who already possess certain characteristics right at the beginning of their study phase which are considered important for professional physicians? Or, should applicants rather be selected if they fit the profile of the selecting university because their study- and job-related interests are aligned with the mission statement of the institution? Perhaps the “Losverfahren” (i.e. the random selection of applicants for a quota of university places), which was abandoned in Germany many years ago, will turn out as the best, cheapest, and fairest selection procedure for motivated study applicants. A scientific analysis of what became of the students with less-than-perfect grades that were admitted at this time could possibly provide the most informative insights to this debate. 

#### Increasing practice orientation

With the changing of the medical licensure act in the year 2002 [Approbationsordnung für Ärzte (ÄAppO), retrieved 31.1.2016], probably all medical schools in Germany have achieved a bigger practice orientation in their curricula in the meantime. It remains unclear though how much practice orientation is needed during the study phase in order to enable students to acquire the competencies required from a licensed medical professional and which practical and communicative skills have to be strengthened accordingly. The National Competency-based Catalogue of Learning Objectives for Undergraduate Medical Educaion (NKLM) [NKLM, retrieved 31.1.2016] that was developed together with the aforementioned groups and associations and adopted in 2015 at the ordinary meeting of the Medical Faculty Association in Kiel could serve as the basis for defining measures to increase practice-orientation [4]. Ultimately, departmens in the individual faculties will have to move away from their historically grown allocation of hours and make a joint effort to convey the contents required for the medical profession. An example for the interdisciplinary use of the NKLM for the development of a longitudinal curriculum for vaccination competency is found in the article by Vogel et al. [5] in this issue. It remains to be seen if the implementation of a competency-base curriculum will lead to changes in capacity legislation in the long-term. Certainly, the implementation of such measures will have to be scientifically monitored to answer the question if an increased practice orientation in the study phase does result in more competent medical professional behaviour and how “practice orientation” can best be defined and realised. First successful validations of newly established competence tests appear to be promising in this regard [6], [7].

#### Strengthening primary care

It has been an institutional and political imperative for many years that, in order to provide adequate care for the rural population, more general practitioners who are willing to work in rural areas are needed. The introduction of a mandatory study section solely focused on primary care has likewise been discussed for several years but it remains unknown as to whether such a measure could contribute to the solution of this problem. In fact, primary care is already one of the most popular and attractive subjects among medical students for specialist training [8]. At the same time, this study also revealed that more than half of the medical students who took part in the survey could not imagine working in towns with a population below 2000 inhabitants. It seems safe to assume that measures other than an increase of the amount of study obligations in this specialty are needed for strengthening the aspect of primary care in healthcare. On the contrary, a forced “overdose” of primary care in the study phase may well lead to a decrease of graduates pursuing further training in this specialty. New concepts for a better distribution of primary medical care are required, which could eventually counteract the lack of doctors in rural areas more effectively. These questions call for more scientific evidence. Overall, a stronger integration of outpatient medical care into the study phase appears to be important as many graduates will later work in this domain. Dedicated educational research projects should investigate if it is mainly primary care that should be strengthened in the study phase or if this may also be true for other disciplines related to primary medical care such as general internal medicine, pediatrics, as well as outpatient medical care in special practices or university outpatient centers [9]. With the GMS Journal for Medical Education [JME, retrieved 31.1.2016], the German Society for Medical Education (GMA) offers a highly visible and well-established platform for critical scientific monitoring and discussion of measures related to the Masterplan 2020.

#### GMS Journal for Medical Education (JME)

The open-access journal of the German Society for Medical Education (GMA), formerly “GMS Zeitschrift für Medizinische Ausbildung (ZMA)”, is now published as “GMS Journal for Medical Education (JME)” with immediate effect. The journal “Medizinische Ausbildung” (“Medical Education”) was first published by GMA in 1984 with the subheading “Forum zur Erforschung der ärztlichen Aus-, Weiter- und Fortbildung” (“Forum for research on medical under- and postgraduate education”). After some intermediate steps in 2005, GMS ZMA eventually turned into an open-access journal in its 22^nd^ year of publication. Since then, GMS ZMA has become the prominent scientific journal for medical education research in the German-speaking community. Since 2010, four bilingual issues appear each year with both German and English full texts. Since 2011, GMS ZMA is MEDLINE^®^/PubMed indexed. Because of a growing international interest, GMS ZMA has become one relevant publication organ for medical education research with new insights and innovative projects for the health professions. The interprofessional and international scope has exceeded the boundaries of the German-speaking community. Because of this growing international perception of the journal, the editorial board and the German Medical Science portal have decided to initiate a name change: From now on the journal will be published under its new name “GMS Journal for Medical Education (JME)”.

## Competing interests

The authors declare, that they have no competing interests.
